# The immune-checkpoint HLA-G/ILT4 is involved in the regulation of VEGF expression in clear cell renal cell carcinoma

**DOI:** 10.1186/s12885-020-07113-8

**Published:** 2020-07-03

**Authors:** Marcela García, Maria Belen Palma, Jerome Verine, Santiago Miriuka, Ana M. Inda, Ana L. Errecalde, François Desgrandchamps, Edgardo D. Carosella, Diana Tronik-Le Roux

**Affiliations:** 1 Chair of Cytology, Histology and Embryology, Faculty of Medical Sciences, UNLP, Buenos Aires, Argentina; 2 LIAN, FLENI-CONICET, Escobar, Argentina; 3 AP-HP, Saint-Louis Hospital, Department of Pathology, Paris, France; 4 CEA, DRF-Francois Jacob Institute, Research Division in Hematology and Immunology (SRHI), Saint-Louis Hospital, 1, avenue Claude Vellefaux, 75010 Paris, France; 5 CIC, Pcia, de Buenos Aires, Argentina; 6 AP-HP, Department of Urology, Saint-Louis Hospital, Paris, France; 7 University of Paris, IRSL, UMRS 976, Paris, France

**Keywords:** HLA-G, ILT4, VEGF, ccRCC, Immune-therapy, Angiogenesis, Lymphangiogenesis

## Abstract

**Background:**

Clear cell renal cell carcinoma (ccRCC), the most aggressive renal cancer, is characterized by early lymph node metastases and bad prognosis. Most therapies targeting advanced or metastatic ccRCC are based, as first-line treatment, on the administration of the vascular endothelial growth factor (VEGF) neutralizing antibody termed Bevacizumab. Despite proven benefits, the expected results were not obtained for the majority of patients. The possibility that an intricate interplay between angiogenesis and immune-checkpoints might exist lead us to evaluate tumor angiogenesis, by means of VEGF expression together with the immune checkpoint HLA-G/ILT4.

**Methods:**

Tumor specimens were obtained from patients from two separate cohorts: One from “Evita Pueblo” Hospital from Berazategui, (Buenos Aires, Argentina) and the second includes patients surgically operated at the Urology Department of Saint-Louis Hospital (Paris, France) with a confirmed ccRCC diagnosis. Immunohistochemistry was performed with specific antibodies directed against HLA-G, VEGF-A, VEGF-C, D240, CD34, ILT4 and Ca-IX. In addition, gene expression levels were measured in a cell line derived from a ccRCC patient by semi-quantitative RT-PCR.

**Results:**

Our results show that the highly vascularized tumors of ccRCC patients express high levels of VEGF and the immune-checkpoint HLA-G. In addition, ILT4, one of the HLA-G receptors, was detected on macrophages surrounding tumor cells, suggesting the generation of an immune-tolerant microenvironment that might favor tumorigenesis. Notably, RT-qPCR analysis provided the first evidence on the transcriptional relationship between HLA-G/ILT4 and the VEGF family. Namely, in the presence of HLA-G or ILT4, the levels of VEGF-A are diminished whereas those of VEGF-C are increased.

**Conclusions:**

In an effort to find new therapeutic molecules and fight against metastasis dissemination associated with the poor survival rates of ccRCC patients, these findings provide the rationale for co-targeting angiogenesis and the immune checkpoint HLA-G.

## Background

Clear cell renal cell carcinoma (ccRCC) is the most common epithelial tumor of the kidney that occurs in adults. It is characterized by malignant epithelial cells with clear cytoplasm and a compact-alveolar or acinar growth pattern interspersed with intricate, arborizing vasculature. ccRCC accounts for approximately 70–80% of renal cancers. Prognosis is generally poor due to insufficient early warning signs and the difficulty to accurately predict tumor aggressiveness [1]. The WHO/ISUP 2016 grading system, which replaces the Fuhrman grading system, is now the more accurate system to classify tumor aggressiveness [2, 3].

The standard care for localized ccRCC remains surgical excision. Patients cannot be treated by radiation nor chemotherapy. Metastases, at diagnosis, were observed for 25–30% of the patients and less than 10% of these patients survive more than five years [4–6]. Recurrence occurs in 20–30% of the patients even after complete nephrectomy of primary tumors.

A major breakthrough in the treatment of advanced or metastatic ccRCC was the introduction, as first-line treatment, of anti-angiogenesis therapies such as the administration of the VEGF-A neutralizing antibody termed Bevacizumab [7, 8]. VEGF-A, the most active isoform promotes the induction of new blood vessels, vascular permeability and cell migration and ultimately may lead to the development of metastases [9–11]. Solid tumors characterized by poorly organized abnormal vessels with altered permeability cannot grow beyond 1–2 mm in diameter without neovascularization [9, 12], demonstrating that tumor growth is dependent on angiogenesis. The degree of intra-tumor angiogenesis in ccRCC might be quantified by measuring the microvessel density (MVD) by means of CD34 expression assessment. CD34 is predominantly regarded as a marker of hematopoietic stem/ progenitor cells. However, CD34 is now established as a marker of several other non-hematopoietic cell types, including vascular endothelium present within newly forming vessels or those just trapped within tumor tissues [5, 13]. Yet, the capacity of the MVD to predict disease recurrence and survival remains controversial.

In recent years, the focus of ccRCC treatment was shifted to therapies targeting the tumor and the immune system simultaneously. Immune-therapies have brought significant improvements in the outcome of some cancer, providing unprecedented clinical benefits [14]. In particular, immune checkpoint (IC) blockade cooperates with other anti-cancer therapies, to increase the effectiveness of therapeutic protocols [15]. IC are crucial for the maintenance of self-tolerance and for the modulation of immune responses in order to minimize tissue damage. They result from the interaction between inhibitory ligand molecules and their receptors present on immune cells. Tumor cells can upregulate checkpoints and escape destruction by the immune system [16, 17].

Recent findings indicated that in the tumor microenvironment, an interconnection between VEGF signaling and immunosuppression might exist, suggesting that the combination of anti-VEGF agents and immune checkpoint blockade might have enhanced synergistic antitumor activity [18].

A recent described checkpoint is the non-classical class I molecule human leukocyte antigen G (HLA-G) [19]. HLA-G was first described to play a major role in foeto-maternal tolerance and tissue transplantation [20]. At present, the checkpoint HLA-G has been found in most tumors analyzed. In particular, high incidence of HLA-G expression has been reported in ccRCC [21–23]. The role of HLA-G as a checkpoint allowing tumor escape has been demonstrated in murine models [24]. HLA-G has a broader inhibitory effect than any other checkpoint since it can block all steps of anti-tumor responses by acting on natural killer (NK) cells, B lymphocytes, T lymphocytes and antigen-presenting cells (APC) through direct interaction with its receptors [19]. Immunoglobulin-like transcript (ILT) 4, one of the HLA-G receptors, was found in ccRCC [22] and non-small cell lung cancer in which promotes tumor progression and metastases by increasing the levels of VEGF-C [25], the best characterized and more efficient growth factor involved in lymphangiogenesis and lymphatic metastases [26–28].

In this context, the aim of this study is to explore the relationship between the immune checkpoint HLA-G/ILT4 and tumor angiogenesis based on the expression of VEGF-A and VEGF-C. The rationale is to determine whether combining conventional anti-VEGF therapies with immunotherapies might improve the outcome of patients with ccRCC.

## Methods

### Patients and tumors

Tumor specimens were obtained from patients from two separate cohorts: One included fifty patients surgically operated from 2006 to 2009 at the Urology Department of “Evita Pueblo” Hospital from Berazategui, (Buenos Aires, Argentina) with confirmed diagnostic of ccRCC, twenty representative of these were presented in this study (Table 1). The second cohort includes patients surgically operated in the Urology Department of Saint-Louis Hospital (Paris, France) and largely described [22, 23]. Both groups of patients underwent radical nephrectomy for ccRCC as first therapy. These renal tumors were classified as ccRCC by experienced uropathologists according to the World Health Organization (WHO) classification of tumors of the kidney [29]. All patients that participated in this study gave their free and informed writing consent. The study was approved by the Institucional Hospital Evita Pueblo committee, the COBIMED (Comité de Bioética y Ética de la Investigación de la Facultad de Ciencias Médicas de la Universidad Nacional de La Plata) and the institutional review board of Saint-Louis Hospital, Paris.

**Table 1:** Clinical and pathological characteristics of the ccRCC patients from the Berazategui Hospital, Argentina. Reference values for Who/ISUP grade, age ranges, MVD index, metastasis, overall survival, local infiltration, tumor size (pT), expression of VEGF-A, HLA-G and ILT4, are provided. The expression levels of markers revealed by IHC were noted as follows: + weak staining; ++ moderate staining; +++ strong staining. Survival, reported as overall survival, is expressed in years considered at 1, 3 or 5 years post-surgery; MVD: Microvessel density index (central and peripheral zone); detection of metastasis: Yes (Y) or Not (N)

Patient	Who/ISUP	Age Range	Metastasis	Survival	VEGF-A	MVD	HLA-G	ILT4	Local infiltration	pT
1	1	70–79	N	< 5	+	36	++	+++	No	T1
2	2	50–59	N	> 5	+	29	++	+++	Capsule	T2
3	2	60–69	Y	< 3	+	14	++	+++	Renal vein	T3
4	2	50–59	N	> 5	++	24	+++	++	Capsule	T1
5	2	50–59	N	> 5	+	38	+++	++	No	T1
6	2	60–69	N	> 5	++	54	+++	+++	Capsule	T1
7	2	60–69	N	> 5	+++	16	++	+++	Capsule	T1
8	2	50–59	N	> 5	++	33	+++	+++	Capsule	T2
9	2	50–59	N	> 5	+	35	+++	+++	Capsule	T1
10	2	50–59	N	> 5	++	34	++	+++	No	T1
11	2	50–59	N	> 5	+	33	++	+	Capsule	T1
12	2	40–49	N	> 5	+	42	++	+	No	T1
13	2	50–59	N	< 5	++	31	+++	+++	Perirenal tissues	T3
14	3	70–79	N	> 5	+++	20	+++	+++	Renal vein	T3
15	3	70–79	Y	> 5	++	16	++	+++	Renal vein	T3
16	3	50–59	N	> 5	+	19	++	+++	Perirenal tissues	T3
17	3	50–59	N	< 3	++	27	++	+++	Perirenal tissues	T3
18	3	70–79	N	> 5	++	26	++	+++	No	T1
19	3	50–59	N	> 5	+	28	++	+	No	T1
20	4	60–69	Y	< 1	++	26	+	+++	Renal vein	T3

### Immunohistochemistry

Immunohistochemistry (IHC) was performed on 4-μm-thick, formalin-fixed, paraffin-embedded tumor tissue sections. The HLA-G labeling was performed with the murine antibody 4H84, an IgG1 recognizing the alpha1 domain of HLA-G isoforms (dilution 1:500, Santa Cruz Biotechnology, Santa Cruz, CA) as described [23]. Its inhibitor receptor ILT4 (dilution 1:50, polyclonal goat antibody, R&D systems) was concomitantly studied. The other primary antibodies used are: anti-VEGF-A (dilution 1:50, clone C-1, mouse monoclonal, Santa Cruz Biotechnology), anti-CD34 (dilution 1:1000, clone BI-3C5, mouse monoclonal, Santa Cruz Biotechnology), anti-VEGF-C (dilution 1:50, rabbit polyclonal, Abcam Inc), the lymphangiogenic growth factor that plays an important role in tumor lymphangiogenesis, anti-D2–40 (dilution 1:20, Mouse monoclonal, Abcam Inc), a specific antibody for lymphatic vessel density (LVD) which reacts with an O-linked sialoglycoproteins found on lymphatic endothelium and anti-carbonic anhydrase (CA)-IX (dilution 1:500, rabbit polyclonal, Novus Biologicals), an enzyme over-expressed in VHL mutated tumors and hypoxic tissues. Detection was performed using DAKO EnVision+ System-HRP (DAKO Corporation, Hamburg, Germany) for 30 min and the reaction was developed using diaminobenzidine, and counter staining with Mayer hematoxylin. Positive and negative controls gave appropriate results for each procedure.

###  Microvessel density (MVD) quantification

MVD was assessed following CD-34 labeling using the criteria of Weidner et al. [30]. A brown CD-34 stained endothelial cell or groups of endothelial cells that clearly separate from adjacent microvessels, tumor cells or other connective tissue elements, were considered as a single countable microvessel. The vessels > 50 μm wide were not considered as neoangiogenesis. Three fields of the most intense vascularization (hotspots) were analyzed for each tumor. The data allow us to obtain the MVD index for each tumor.

### Cell lines

The RCC7 cell line used in this study derives from a patient with ccRCC [31] and was kindly provided by Anne Caignard. The cell line is routinely tested for mycoplasma contamination by using the LookOut®Mycoplasma PCR detection kit. These cells express VEGF-A and VEGF-C but not HLA-G nor ILT4. RCC7 cells expressing HLA-G and ILT4 were obtained after lentiviral transduction of their respective cDNA in plasmid pWPXL (Addgene plasmid # 12257). The M8 HLA class I-positive melanoma cell line expressing HLA-G was previously described [32].

### Production of lentiviral particles containing HLA-G or ILT4 cDNAs

HLA-G1 cDNA was introduced into the plasmid pWPXL (10,510 bp), between the BamH1 and NdeI sites, just 3 ‘of the EF-1α promoter. This was followed by a “red” variant of the GFP (*Aequorea victoria* green fluorescent protein jellyfish), named Neptune that has been brought under control of the CMV promoter. The ILT4 cDNA (1797 bp) was introduced into the plasmid pWPXL between the Ml1 and NdeI sites, just 3 ‘of the EF-1α promoter. These 2 plasmids were used to produce lentivirus WPXL ΔU3 SIN, envelope VSV-G, OGM group II, class 2 at 1.20E + 08 TU and 1.00E + 08 (Transduction Unit) / ml respectively at the Plateforme Vecteurs Viraux et Transfert de Gènes (VVTG), SFR Necker, US 24, UMS 3633, Paris.

Lentivirus (10^7^ pfu/ml) expressing either HLA-G or ILT4 were added to RCC7 cells (10.000 cells/well in 12-well plates) at a multiplicity of infection (MOI) of 5. Cells were incubated overnight at 37 °C in a humidified incubator (5% CO_2_). Media containing lentiviral particles were then removed from wells. Cells were washed and cultured in fresh medium (DMEM + 10% FBS). To expand the culture, cells arrived at 90% of confluence were plated in a T-25 cm^2^ flask. The efficiency of transduction (approximately 30%) was determined using fluorescence microscopy and flow cytometry. Transduced cells were then sorted using BD FACSAria III (BD Biosciences-US) to obtain 95% of purity.

### RNA extraction

Total RNA was isolated from tissue sections manually crushed in Trizol™ Reagent (LifeTechnologie, ref. 15,596,026). After chloroform separation, the RNA was purified using miRNeasy mini Kit (Qiagen, ref. 217,004) according to the manufacturer’s instruction, with a DNase extra step treatment (Qiagen, ref. 79,254). The concentration and purity of RNAs was assessed using a Nanodrop spectrophotometer.

### Real time RT-PCR

Total RNA was used as template for cDNA production using High Capacity cDNA Reverse Transcription kit (Applied Biosystems, USA). For cDNA amplification Power SYBR Green PCR Master Mix (Applied Biosystems, USA) was added and the mixture was poured up into a MicroAmp Optical 96-well Reaction Plate (Applied Biosystems, USA) that contained primers for the different genes (forward and reverse respectively):

HLA-G: 5′-GGAAGAGGAGACACGGAACA; 5′ - CCTTTGTTCAGCCACATTGG;

ILT-4: 5′-GCATCTTGGATTACACGGATACG; 5′-GTGGGTTTTGGGTAGGCTC;

VEGFA: 5′- CTTGCCTTGCTGCTCTACC; 5′- CACACAGGATGGCTTGAAG;

VEGF-C: 5′-ATGTGTGTCCGTCTACAGATGT; 5′-GGAAGTGTGATTGGCAAAACTGA;

Actin B: 5′-TCCTGTGGCATCCACGAAACT; 5′-GAAGCATTTGCGGTGGACGAT.

Thermal cycling was performed using ABI-7000 (Applied Biosystems, USA) according to manufacturer’s instruction with an initial denaturation at 95 °C for 10 min, 40 cycles at 95 °C for 15 s, and 60 °C for 1 min. Values of cycle threshold (Ct) were used for calculations of fold changes in mRNA abundance using 2^-ΔΔCt^ method.

### Statistical analysis

The expression levels were analyzed by ANOVA, followed by Kramer Multiple Comparisons Test (using 95–99% confidence interval).

## Results

### Clinicopathologic characteristics of patients with ccRCC

A retrospective study was performed on twenty representative patients (Table 1) from a total of fifty included in the Argentinian cohort, after obtaining ethical committee clearance.

The tumors derived from fifteen men and five women, of which 11 patients were 50–60 years old. This proportion is consistent with what is expected for ccRCC patients since this neoplasm is highly aggressive and affects relative young people [5]. The WHO/ISUP grade of the 20 patients are as follows: Grade 1:1; Grade 2:12; Grade 3: 6 and Grade 4:1, being grade 1 the least aggressive type and grade 4 the most aggressive. Their clinical history revealed that six patients did not survive 5 years post-surgery, of whom three developed metastases (Table 1). The reason of patients’ death within 5 years was due to: cardiac decompensation (patients 1 and 17), pulmonary and cerebral metastases (patient 20), breast cancer (patient 14) and tumor progression (patients 3 and 15). Local infiltration data and tumor size status (pT) are also shown (Table 1). The correlation analysis revealed that when the WHO/ISUP grade is high, usually the pT status is high. For WHO/ISUP group 1 there is one patient with pT1. For WHO/ISUP group 2, 67% of patients are pT1, 17% are pT2 and 17% are pT3. For WHO/ISUP group 3, 67% of patients are pT3 and 33% are pT1. For WHO/ISUP group 4 there is one patient with pT3 (Table 2), being pT1 the smallest tumor size and pT4 the largest.

**Table 2:** Correlation analysis between WHO/ISUP grade and tumor size status (pT). The table shows relative frequencies obtained from the data of Argentinian patients

WHO/ISUP	T1	T2	T3	Total
1	1,00	0,00	0,00	1,00
2	0,67	0,17	0,17	1,00
3	0,33	0,00	0,67	1,00
4	0,00	0,00	1,00	1,00
Total	0,55	0,10	0,35	1,00

### Immuno-histochemical evaluation of tumor angiogenesis in ccRCC

To evaluate angiogenesis, we first carried out IHC on the twenty ccRCC samples using specific antibodies for VEGF and CD34. The results show that all tumors samples expressed VEGF-A in at least one area of the tumor. For comparative purpose with the subsequent genes analyzed in this study, the expression was scored according to stain intensity: weak, moderate or high (Table 1). Of note, high levels of VEGF-A do not correlate with the highest WHO/ISUP grade.

**Fig. 1:**
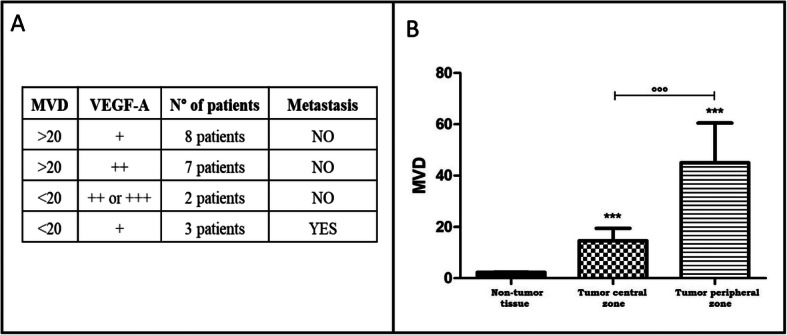
Measure of MVD index. **a** Correlation between MVD index and VEGF-A expression. **b** MVD Average index in tumor and adjacent non-tumor regions. Normal: adjacent non-tumor tissue; central: tumor central zone; peripheral: tumor peripheral zone. The standard deviation is shown

To test whether the angiogenic capacity of a tumor increases parallel to its microvasculature, antibodies directed against the CD34 antigen were used to calculate the MVD index. A brown CD-34 stained endothelial cell or groups of endothelial cells that clearly separate from adjacent microvessels, tumor cells or other connective tissue elements, were considered as a single countable microvessel. According to the results, tumor samples were divided into two groups: group 1, with more than 20 microvessels per field and group 2, with less than 20 microvessels per field. The MVD tumor mean was 29.05 microvessels/ field. Only four patients belong to the second group confirming that ccRCC samples have important tumor neoangiogenesis. Notably, in most patients with MVD > 20, VEGF-A levels are low (+ or ++) and those with MVD < 20, VEGF-A are high (Fig. 1a). This is no longer the case for patients that have developed metastases for whom levels of both, CD34 and VEGF-A, were low. We observed high MVD index in the peripheral tumor area compared with the central region, but all tumor regions have significantly higher MVD index compared with normal adjacent renal tissue (Fig. 1b). It is likely that studying the difference between the intra- and peritumoral MVD might lead in the future to a better assessment of prognosis.

### Immuno-histochemical survey of the immune checkpoint HLA-G/ILT4

Since a possible interplay between angiogenesis and immune-checkpoints has lately been evoked as a novel strategy to improve the success of cancer treatments, we ought to evaluate the potential co-expression of VEGF-A with the immune checkpoint HLA-G/ILT4.

First, tumor samples were analyzed by IHC with antibody 4H84, which recognizes the alpha1 domain of the seven reported HLA-G isoforms. IHC staining was detected in at least one area of all samples. This expression was variable among patients and the different areas of the samples in accordance with previous studies [23]. The staining was membranous and cytoplasmic and was annotated according to the stain intensity: weak, moderate or high (Table 1).

**Fig. 2:**
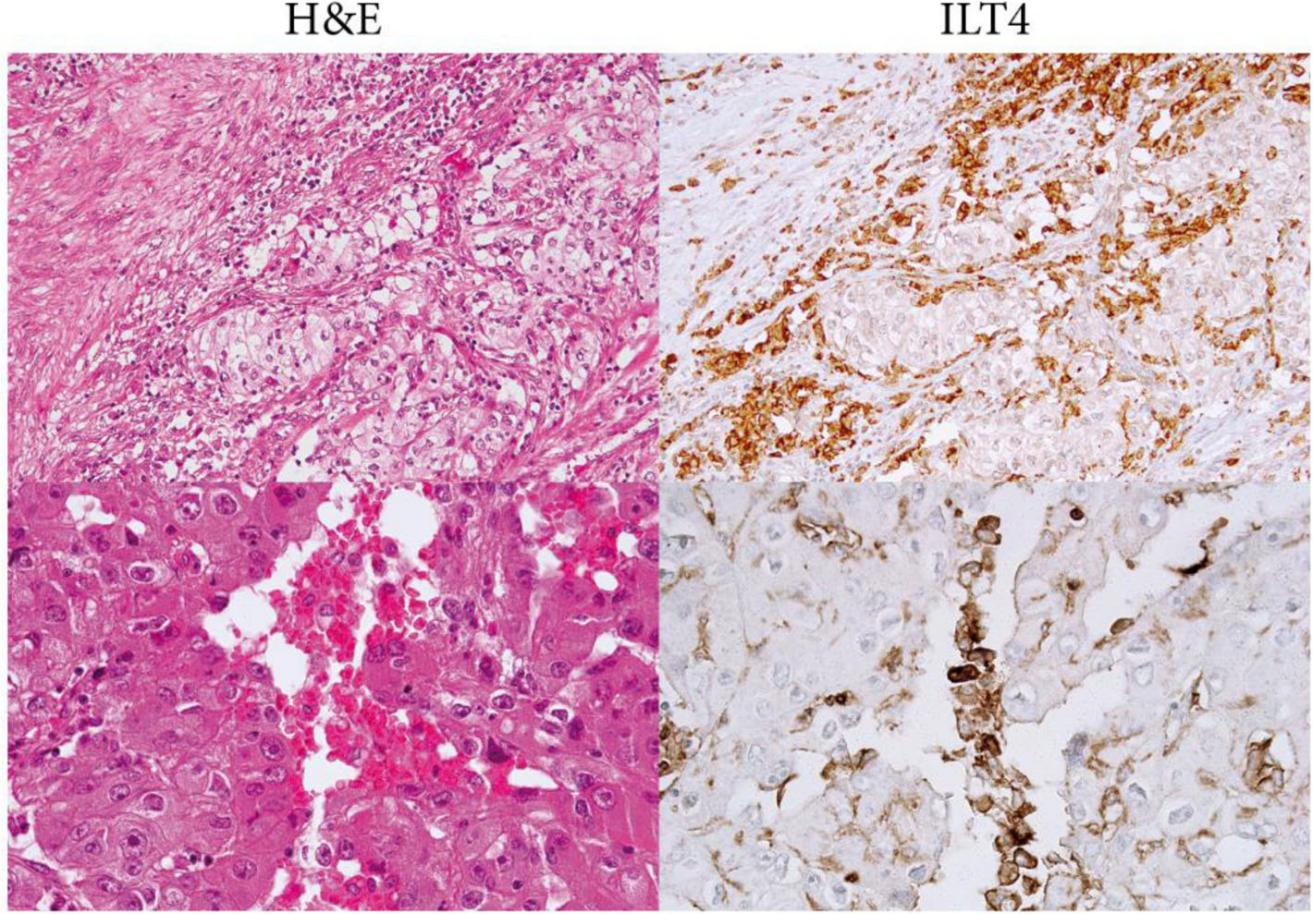
Representative IHC staining of ILT4 in tumor tissues of two patients with ccRCC (H&E and immunoperoxidase stains are also shown). No label was detected in normal adjacent tissues

Next, we assessed the expression of ILT4, the HLA-G receptor. All tumor samples from the twenty Argentinian patients were positively labeled (Table 1). The stain was detected on some stromal macrophages, plasma cells and infiltrating lymphocytes. Fig. 2 shows two representative examples. Together, the high expression of HLA-G and its receptor ILT4 is consistent with the generation of an immune-tolerant microenvironment as previously described [17, 33].

### Marked expression heterogeneity of ccRCC specimens

The retrospective study clearly demonstrates high expression of VEGF-A, HLA-G and ILT4, but does not inform us whether these molecules are co-expressed in the same areas of the tumor. Therefore, we have performed an IHC study. Due to the potential heterogeneity of expression, tumors obtained from patients from the French Saint Louis Hospital cohort [22] were cut in several pieces (3 to 6 according to the tumor size). Since these tumors were previously asses for the expression of HLA-G (23), we purposely chose HLA-G-positive and HLA-G-negative tumors in order to evaluate whether correlations might exist with the other markers (VEGF-A, CD34, VEGF-C, D2–40, ILT4 and CA IX).

**Fig. 3:**
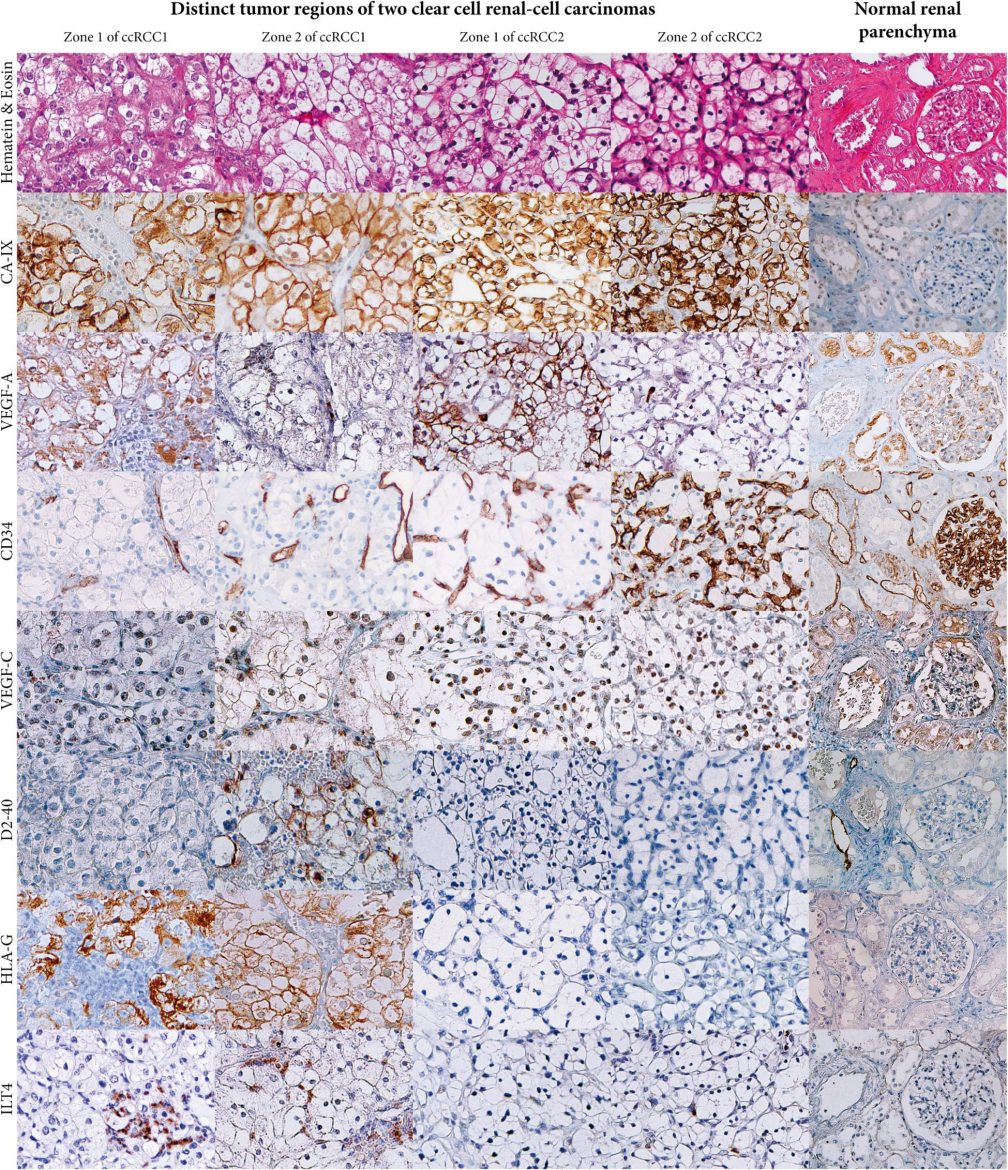
Immunohistochemical labeling for CA-IX, VEGF-A VEGF-C, CD34, D2–40, HLA-G and ILT4 in distinct tumor regions of two representative clear cell renal-cell carcinoma samples. Expression is observed as a dark brown color. (H&E and immunoperoxidase stains are also shown)

The IHC results reveal positive staining for the four markers VEGF-A, CD34, HLA-G and ILT4 which confirms the retrospective results. Consistent with our previous studies [23], HLA-G and ILT4, were not detected on normal parenchyma. Some tumor regions exhibited strong immunostaining with anti-VEGF-A whereas no label was found for HLA-G or ILT4 (Fig. 3). Similarly, some tumor regions exhibited strong immunostaining with anti-HLA-G or anti-ILT4 whereas no label was found for VEGF-A. In addition, the IHC staining revealed that regions showing strong labeling for D2–40 were also highly stained with antibodies directed against HLA-G and ILT4. Moreover, no concordance was found between these expression profiles and those of VEGF-C. The immunostaining with anti-carbonic anhydrase (CA)-IX was strongly observed in all tumor samples, which would correlate with a pseudo-hypoxic tumor status that is commonly present in this neoplasia and is associated with the VHL mutation, consistent with previous literature reports [34, 35].

Altogether, the IHC study reveals significant positive labeling of angiogenic and immune-checkpoints markers in ccRCC samples. However a clear heterogeneity was observed with no perceptible association among markers. Given this heterogeneity and the variety of factors that can regulate their expression, a more detailed molecular analysis appears to be necessary to better understand the basis of ccRCC.

### The immune-checkpoint HLA-G/ILT4 is involved in the expression of VEGF-A and VEGF-C

In view of the heterogeneity of the IHC results, we aimed to analyze more specifically the influence of HLA-G and ILT4 on the expression of VEGF genes at the molecular level. To overcome the difficulty that represents analyzing transcriptional regulation of genes in a heterogeneous tumor microenvironment, the analysis was performed in the cell line RCC7, which constitutes a valuable cellular model for ccRCC [31].

**Fig. 4:**
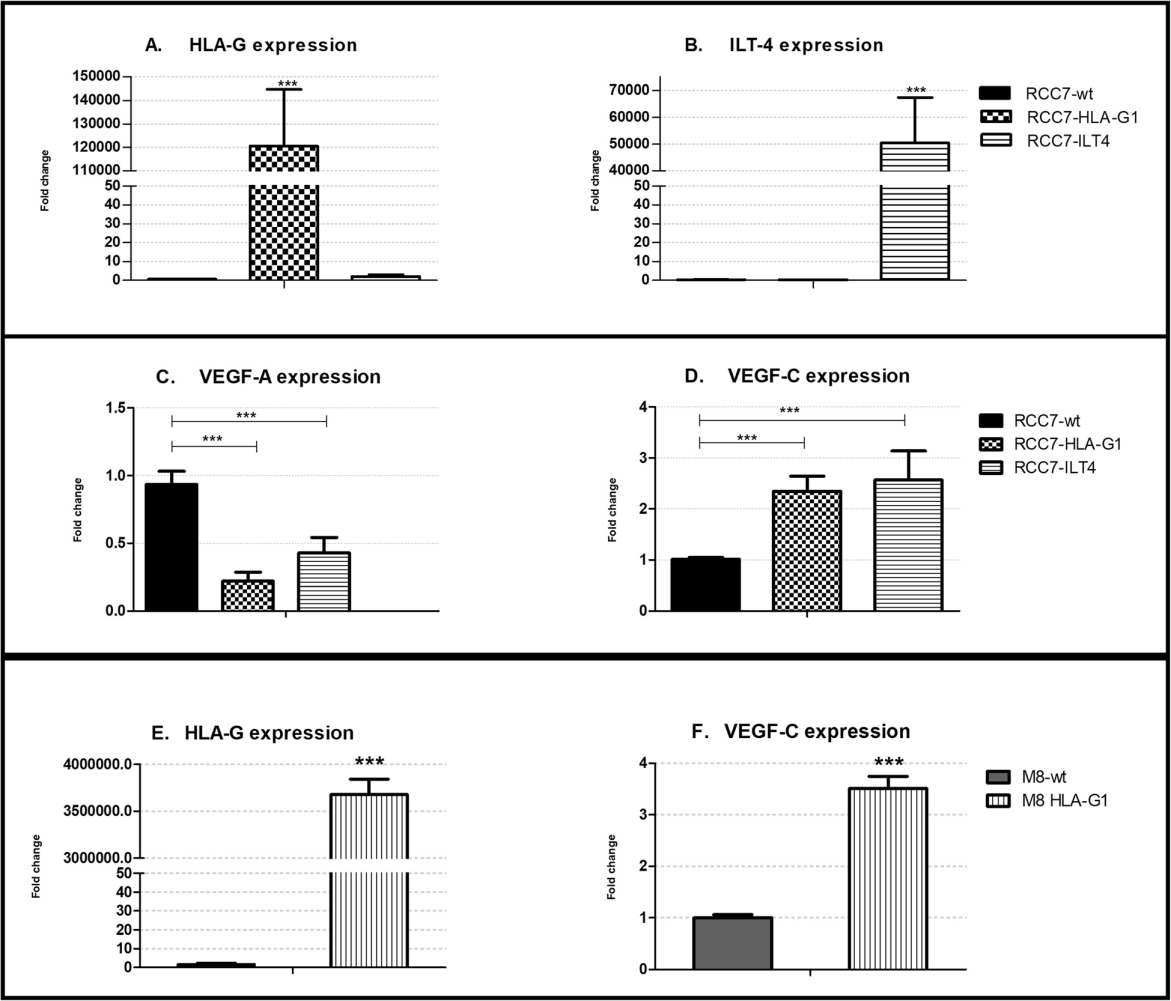
Effect of the checkpoint HLA-G/ILT4 on the expression of VEGF genes. RNA prepared from RCC7-wild type (wt), RCC7-HLA-G1, RCC7-ILT4, M8- wild type (wt) and M8-HLA-G1 cell lines were subjected to semi-quantitative RT-PCR using specific primers. The symbol (***) shows that differential expression is statistically significant (*p* < 0,01). **a** HLA-G1 expression in the three cell lines; **b** ILT4 expression in the three cell lines; **c** VEGF-A expression in the three cell lines; **d **VEGF-C expression in the three cell lines ;  **e** HLA-G expression in M8 (wt) and M8-HLA-G1 and **f **VEGF-C expression in M8-(wt) and M8-HLA-G1.

The expression levels of HLA-G, ILT4, VEGF-A and VEGF-C were analyzed by RT-PCR with specific primers. The results showed that VEGF-A and VEGF-C are highly expressed in the RCC7 cell line whereas the immune checkpoint HLA-G/ILT4 is not. We therefore transduced RCC7 cells with lentivirus carrying the cDNA of HLA-G or ILT4 respectively. The expression of HLA-G and ILT4 in these transduced cells was first confirmed by RT-PCR (Fig. 4a and 4b). Then, to determine whether the presence of HLA-G or ILT4 could modulate the expression of the VEGF genes, we measured VEGF-A and VEGF-C mRNA levels in the three cell lines (RCC7, RCC7-HLA-G1 and RCC7-ILT4). The results show a 70% decrease in the levels of VEGF-A in the presence of HLA-G and a 60% decrease in the RCC7 cells expressing ILT4. In sharp contrast, the levels of VEGF-C were 2,5-fold higher in the RCC7 cells expressing HLA-G and ILT4 than those of controls (Fig. 4c and 4d).

Altogether, a significant increase in the levels of VEGF-C (*p* < 0,01) was observed in cells expressing HLA-G or ILT-4 whereas the VEGF-A expression levels were weaker (p < 0,01) in cells expressing HLA-G or ILT-4 than in wild-type RCC7.

To determine whether the effect of HLA-G was cell-dependent, we measure the expression of VEGF-C in the melanoma cell line M8 expressing HLA-G [32]. The results demonstrate that VEGF-C is induced also in M8 cells (Fig. 4e and f), proving the direct role of HLA-G on VEGF-C expression.

## Discussion

The standard care for advanced or metastatic ccRCC patients, when nephrectomy is not efficient enough, is to use anti-angiogenic therapies aimed to hinder blood supply for the tumor, mainly by the administration of anti-VEGF-A monoclonal antibody Bevacizumab [36]. Despite proven benefits, these treatments have not given fully expected results in the majority of patients [7, 37] emphasizing the need to design more effective protocols to control ccRCC proliferation and increase survival of patients. Within this context, we evaluated the relationship between angiogenesis and immune checkpoints.

To evaluate angiogenesis in patients with ccRCC, we sought to determine first whether a correlation exists between the tumor’s grade, VEGF-A positivity and MVD calculated by the CD34 labeling. We observed that all the tumors analyzed were positive for VEGF-A expression in at least one area of the tumor and have an important MVD. Nevertheless, tumor’s grade was found to be independent of both markers. In addition, we found a negative correlation between VEGF-A and the MVD index. When VEGF-A expression was high, the MVD index was low (less than 20 microvessels per field), whereas when the VEGF-A expression was low, the MDV index was high. This unexpected situation might be explained by the fact that a well-irrigated tumor region (ie MVD high) does not need the formation of new vascular vessels, consequently VEGF-A expression by tumor cells decreases. Conversely, when the tumor region is poorly irrigated (ie MVD low), this low oxygen (hypoxic) microenvironment will induce the expression of the hypoxia-inducible factor (HIF) by tumor cells, which directly stimulates the expression of VEGF-A [38]. This is no longer the case for patients that have developed metastasis whose levels of both, MVD and VEGF-A, are low. In addition, a higher MVD was found in the peripheral zone compared with the central region. It is likely that the peripheral tumor zone have the most metabolically active tumor cells, which are continuously expanding. Such a process would require good oxygenation provided by intense neo-angiogenesis. In addition, we observed high levels of CA-IX that might correspond with pseudo-hypoxic areas as previously reported in literature (34, 35). It still remains unclear whether normal vessels and tumor vessels show the same immune-reactivity with various antibodies [39].

In the search of complementary angiogenic strategies to improve the clinical response of ccRCC patients, we have asked whether combining conventional anti-angiogenic therapies with blockade of the immune checkpoint HLA-G/ILT4 might be associated with a significant improvement in durable response rate since previous studies suggested an intricate interplay between angiogenesis and immune-surveillance [38]. To this end, we have simultaneously analyzed the expression of VEGF genes with the immune-checkpoint HLA-G/ILT4.

The transcriptional analysis provided the first evidence on the relationship between the HLA-G/ILT4 and the VEGF family. We demonstrated in the ccRCC7 cell line engineered to overexpress HLA-G or ILT4, that in the presence of one of these two molecules, the levels of VEGF-A are reduced whereas those of VEGF-C increase. This is consistent with a previous report showing that in a non-small cell lung cancer (NSCLC), ILT4 plays an important role in promoting tumor growth and metastases by the upregulation of VEGF-C expression [22, 40–42] which in turn induces the abnormal growth of peripheral lymphatic vessels and increases their permeability facilitating lymphatic metastasis. ILT4 and VEGF-C were also shown to be involved in the epithelial-to-mesenchymal-transition (EMT) process, which endows transformed endothelial cells with the ability to invade and disseminate [43, 44]. Moreover, the regulation of VEGF-C by HLA-G was shown to be cell-independent since the levels of VEGF-C also increase in a melanoma cell line when transfected with HLA-G. As ILT4, HLA-G was associated with tumor metastases and poor survival in an animal model of ovarian cancer [45]. Altogether, our results further suggest that the presence of the immune-checkpoint HLA-G/ILT4 bring forth an effective signal transmission to form an immunosuppressive microenvironment and enhance the formation of new tumor lymphatic vessels increasing therefore the metastatic capacity of tumor cells [28].

## Conclusions

The data reported here may be valuable to control tumor growth and improve outcomes for patients with ccRCC. Even though the different regulatory cascades which simultaneously involve either VEGF-A or VEGF-C are still to be clearly determined, we propose considering the establishment of protocols associating the anti-VEGF-A antibody Bevacizumab, which should prevent the increase in the levels of VEGF-A, with the blockade of HLA-G, which should prevent tumor progression and metastases by decreasing the levels of VEGF-C and re-activate the immune system. We believe that the design of these combined therapies would be the new direction to be attempted in the future to improve the effectiveness of treatment strategies for patients with ccRCC.

## Data Availability

Data generated or analyzed during this study are included in this published article. Extra data and materials from Argentinian patients which were not included in this manuscript, were not used to draw the conclusions but are available on request from the corresponding author.

## Abbreviations

ccRCC: Clear cell renal cell carcinoma
WHO/ISUP: World Health Organization / International Society of Urologic Pathologists
VEGF: Vascular endothelial growth factor
HLA-G: Human leukocyte antigen G
ILT4: Immunoglobulin-like transcript 4
MVD: Microvessel density
PCR: Polymerase chain reaction
